# Quantification of Chemotherapy Drug Wastage and Incurred Financial Loss in Paediatric Cancer Care: A Cross-Sectional Study at a Tertiary Care Public Hospital in India

**DOI:** 10.7759/cureus.52076

**Published:** 2024-01-11

**Authors:** Shweta Ghate, Swati Patil, Neha Kadhe, Rutuja Fulsoundar, Sudhir Pawar

**Affiliations:** 1 Pharmacology and Therapeutics, Lokmanya Tilak Municipal Medical College & Hospital, Mumbai, IND; 2 Clinical Pharmacology, Seth Gordhandas Sunderdas Medical College, Mumbai, IND

**Keywords:** rounding of doses, cost, drug vial optimisation, vial sharing, vial size, health economics

## Abstract

Aim: The evolving chemotherapy landscape continually introduces effective agents, but escalating costs call for an evaluation of drug wastage and financial consequences to enhance resource utilization. This study seeks to estimate chemotherapy drug wastage and its economic loss in paediatric cancer care.

Methods: In this cross-sectional study of paediatric cancer patients receiving parenteral chemotherapy, we evaluated both the drug used and wasted during each administration. The monetary value of drug loss was calculated using the formula: Cost = Proportion of drug wasted X Cost of drug vial.

Result: A total of 100 paediatric cancer patients who received 140 parenteral drug administrations of 22 chemotherapy drugs were studied. The total amount of drug procured was 25,515 mg, out of which 5,004.9 mg were wasted. Wastage amounted to 19.61% of the procured drugs in varying proportions. The total estimated cost of chemotherapy stood at 110,143.1 INR (1,328.7 USD), with cost wastage accounting for 31,929.95 INR (385.19 USD), equivalent to 28.98% of the total expenditure. Notably, doxorubicin 112.2 mg (37.4%) exhibited the highest drug wastage, followed by cytarabine 280 mg (35%) and l-asparaginase 83,400 IU (26.9%), primarily prescribed for acute lymphocytic leukaemia. Cytarabine resulted in the highest financial loss. Dose rounding occurred in 22 cases (15.71%), while vial sharing was observed in only five cases (3.57%) during drug administrations. Methotrexate, doxorubicin, and cytarabine doses never matched the available vial sizes.

Conclusions: In resource-limited healthcare settings, implementing centre-specific measures, such as vial sharing and drug categorization, can reduce drug wastage and financial losses. Evaluating the viability of optimizing vial sizes and producing multidose vials is essential.

## Introduction

The incidence of childhood cancers in India is alarmingly high, with 75-150 childhood cancers per million children, comprising about 20% of all paediatric cancers worldwide [1]. As per GLOBOCAN 2020, India records one million new cancer diagnoses annually, of which 3% occur in children [2]. However, life-saving cancer treatments are expensive and can put a significant financial burden on families, leading to further disruptions in family dynamics. In India, patients bear almost 68% of their healthcare expenditure, which is much higher compared to the world average of 18% where the majority is under insurance coverage. Additionally, medicines account for 70% of the out-of-pocket expenditure on healthcare by individuals and families in India, making it a significant barrier to healthcare access, particularly for those with a lower socioeconomic status [3].

As per current chemotherapy practices, the dosing of drugs is determined by several factors, including the type of cancer, stage, treatment protocol, weight or body surface area of the patient, age, comorbidities, and general health of the patient. Therefore, there may be variations in chemotherapy drug doses among patients using the same protocol for the treatment of the same cancer [4]. However, most chemotherapy formulations are available as single-dose packages, which can lead to drug wastage if the amount of drug required does not match the amount of the drug in the vial. This is mainly caused by administering chemotherapy drugs from vials containing larger amounts than required and not using the remaining portion of the vial, leading to drug wastage. This contributes to the increasing overall burden of cancer care [5]. However, limited vial size options and drug stability can restrict the potential of vial sharing between patients.

According to studies conducted by Gopisankar et al. [6] and Truong et al. [7], drug wastage and its economic implications significantly add to the cost of cancer care without providing any incremental value to patients. Research in this field has suggested options such as vial sharing, rounding of doses, and batching of patients as per pathology to curb wastage and decrease costs [8].

Most studies on drug wastage for chemotherapy drugs have focused on their use in adult oncology care. Hence, this study aimed to estimate drug wastage specifically in the treatment of paediatric malignancies at our hospital. Additionally, the economic impact of this wastage was estimated, and existing measures (if any) for decreasing drug wastage were noted.

This paper was posted as a preprint on Authorea in October 2023 (https://doi.org/10.22541/au.169777817.76236390/v1) [9].

## Materials and methods

The study was conducted as a single-centre, cross-sectional, observational study in the paediatric oncology day care unit of a tertiary care hospital in India from November 2019 to May 2021. A sample size of 100 patients was targeted based on patients attending the daycare unit during this period. Ethical approval was obtained from the Institutional Ethics Committee (IEC) with the IEC number IEC/381/19, and written informed consent and assent were obtained from all participants before enrolling them in the study.

The study included paediatric cancer patients (less than 18 years of age) who were scheduled to receive parenteral chemotherapy drugs. Patients whose chemotherapy details were recorded once were excluded from the study. The details were recorded based on the information obtained from the patient and their medical record. The information recorded included the chemotherapy drug, dose (mg/m^2^), total calculated dose, formulation details, actual drug consumed (mg), drug wasted (mg), the total cost of the drug (INR (USD)), cost of the wasted drug (INR (USD)), and measures taken to reduce drug wastage (if any), such as rounding off the dose or sharing of the vial. All this information was recorded on a case record form. The cost of one unit was obtained from the hospital pharmacy, outside pharmacies, or NGOs. If provided by NGOs, the retail price for that brand was noted by the pharmacy, and an expense assessment was conducted. The monetary value of drug loss was calculated using the formula: Cost = Proportion of drug wasted X Cost of drug vial.

Our independent analysis of drug wastage included the following scenarios: 1) calculation of the unused dose by subtracting the prescribed dose from the total amount of drug in the vial in mg; 2) estimation of the total cost of the drug in INR and the cost of the wasted drug; and 3) determination of the frequency of sharing vial or rounding off dose if applicable.

We also estimated whether the prescribed doses for different chemotherapy drugs matched the vial size availability at our hospital pharmacy and outside pharmacies as per the Current Index of Medical Specialities (CIMS) 2022.

Statistical analysis: All collected data were entered into Microsoft Excel 2016. Continuous variables were expressed as mean ± standard deviation (SD), and categorical variables were expressed as percentages and frequency. The percentage of drug wastage was determined at a 95% confidence interval.

## Results

Our analysis included 100 patients who received 140 parenteral chemotherapy drug administrations of 22 different drugs. The mean (±SD) age of patients was 6.67 (±3.63) years, with a male predominance of 67 (67%) compared to females, who were 33 (33%).

Hematological malignancies were observed in 89 (89%) of our study population (n=100) compared to solid tumors, which were observed in 11 (11%) (Table 1).

**Table 1:** Percentage of cancer distribution (n=100) n=Frequency

Broad Category of Malignancy	Type of Malignancy	Frequency n (%)
Haematological malignancies (n=89)	Acute lymphocytic leukaemia	74 (74%)
	Acute myeloid leukaemia	7 (7%)
	Hodgkin’s lymphoma	5 (5%)
	Non-Hodgkin lymphoma	2 (2%)
	Langerhans cell histiocytosis	1 (1%)
Solid tumour (n=11)	Germ cell tumour	2 (2%)
	Rhabdomyosarcoma	2 (2%)
	Clear cell sarcoma of the kidney	1 (1%)
	Primitive neuroectodermal tumour	1 (1%)
	Medulloblastoma	1 (1%)
	Neuroblastoma	1 (1%)
	Optic glioma	1 (1%)
	Retinoblastoma	1 (1%)
	Wilms tumour	1 (1%)

We categorized the 140 prescriptions recorded as frequently prescribed drugs (i.e., drugs with >3 prescriptions) and infrequently prescribed drugs. Of the frequently administered drugs (n=119), L-asparaginase 31 (22.14%) and vincristine 29 (20.71%) were the most commonly prescribed. Among the 22 infrequently administered drugs (drugs with ≤3 prescriptions), etoposide 3 (2.14%) and HD cytarabine 3 (2.14%) were the most commonly prescribed.

All parenteral chemotherapy drugs studied were available either as a single-use liquid 73 (52.14%) or powder 67 (47.85%) formulation.

Drug Wastage

Out of the total amount of drug procured for 140 administrations, which was 25,515 mg, 5,004.9 mg of the drug were wasted, accounting for an overall wastage of 19.61% of the drug procured. The wastage for individual drugs ranged from 9.6% to 37.4% in our study, as shown in Table 2 and Table 3.

**Table 2:** Drug wastage for frequently prescribed drugs (n=119) *: Drug amount in IU; Total number of frequently prescribed drugs, n=119; CI: Confidence interval

Drug name	Frequency of drug administrations (n)	The total amount of available drug in vial (mg)	Total amount of drug consumed (mg)	Total amount of drug wastage (mg)	Percentage of drug wastage (95% CI)
Doxorubicin	6	300	187.8	112.2	37.4 (16.67-58.12)
Cytarabine	8	800	442.5	280	35 (0-62.5)
L-Asparaginase*	31	305,000	222,200	83,400	27.34 (20.27-33.52)
Daunorubicin	8	160	121.4	38.6	24.13 (13.68-28.48)
Methotrexate (IT)	26	390	300	90	23.08 (2.50-19.3)
Vincristine	29	44	36.1	8.2	18.63 (3.73-29.71)
Cyclophosphamide	6	4,900	4,015	885	18.06 (5.96-34.86)
Methotrexate (IV)	5	10,000	9,040	960	9.6 (20.06-26.08)

**Table 3:** Drug wastage for infrequently prescribed drugs (n=22) Total number of infrequently prescribed drugs, n=22

Drug name	Frequency of drug administrations (n)	Total amount of available drug in vials (mg)	Total amount of drug consumed (mg)	Total amount of drug wastage (mg)	Percentage of drug wastage (%)
Irinotecan	1	100	15	85	85
Fludarabine	1	50	16	34	68
Etoposide	3	300	100	200	67
Cisplatin	2	100	40	60	60
Vinorelbin	1	50	20	30	60
Vinblastine	1	10	4.2	5.8	58
HD cytarabine	3	5000	2835	2165	43.3
Arsenic trioxide	1	3	2.5	0.5	17
Idarubicin	1	5	4.4	0.6	12
Gemcitabine	1	1000	950	50	5
Bortezomib	1	1	1	0	0
Carboplatin	2	300	300	0	0
Dactinomycin	2	2	2	0	0
Ifosfamide	1	2000	2000	0	0

Our analysis revealed that doxorubicin 112.2 mg (37.4%) had the highest amount of drug wastage, followed by cytarabine 280 mg (35%) and L-asparaginase 83,400 IU (26.9%), which were prescribed for acute lymphocytic leukaemia.

For infrequently prescribed drugs, single administrations of irinotecan for primitive neuroectodermal tumours and fludarabine for acute myeloid leukaemia resulted in 85 mg (85%) and 34 mg (68%) of drug wastage, respectively. However, no drug wastage was observed for bortezomib, carboplatin, dactinomycin, and ifosfamide, as shown in Table 3.

Economic Burden of Drug Wastage

All drugs used in our study were procured from the NGO, and their costing was noted from the hospital pharmacy. The total expenditure for drug procurement was 110,143.1 INR (1,328.7 USD), out of which 31,929.95 INR (385.19 USD) was the cost of wasted drugs, amounting to 28.98% of the economic loss, as shown in Table 4.

**Table 4:** Cost for wastage for frequently prescribed drugs (n=119) *: Drug amount in IU; Total number of frequently prescribed drugs, n=119; CI: Confidence interval

Drug name	Frequency of drug administrations (n)	Total cost of drug INR (USD)	Total cost of drug wasted INR (USD)	Percentage of cost of drug wastage (95% CI)
Cytarabine	8	1600 (19.30)	681 (8.22)	42.56 (18-62.5)
Doxorubicin	6	9240 (111.47)	3301.36 (39.83)	35.73 (13.72-57.74)
L-Asparaginase*	31	43400 (523.55)	12054 (145.41)	27.77 (21.33-34.21)
Daunorubicin	8	3184 (38.41)	768 (9.26)	24.12 (15.72-32.52)
Methotrexate (IT)	26	1254.5 (15.13)	289.27 (3.49)	23.06 (20.05-26.07)
Vincristine	29	1720 (20.75)	328 (3.96)	19.07 (11.38-24.83)
Cyclophosphamide	6	935 (11.28)	186.25 (2.25)	19.92 (5.97-34.87)
Methotrexate (IV)	5	14400 (173.71)	1382.4 (16.68)	9.6 (5.97-34.87)

For infrequently administered drugs, the single administration of irinotecan and fludarabine resulted in a financial loss of 3,305 INR (39.87 USD) and 6,204 INR (74.84 USD), respectively. The financial loss with wastage of all infrequently prescribed drugs is provided as a tabulation in Supplementary Table 5.

**Table 5:** Supplementary table: Percentage of cost wastage of infrequently prescribed drugs (n=22) Total number of infrequently prescribed drugs, n=22

Drug name	Frequency of drug administrations (n)	Total cost of drug INR (USD)	Total cost of drug wastage INR (USD)	Percentage of cost of drug wastage (%)
Irinotecan	1	3889 (46.91)	3305 (39.87)	85
Fludarabine	1	9124 (110.07)	6204.3 (74.85)	68
Etoposide	3	600 (7.24)	400 (4.83)	67
Cisplatin	2	1022 (12.33)	613.2 (7.40)	60
Vinorelbin	1	1000 (12.06)	600 (7.24)	60
Vinblastine	1	250 (3.02)	145 (1.75)	58
HD cytarabine	3	3840 (46.32)	1310.4 (15.81)	34.12
Arsenic trioxide	1	160.62 (1.94)	26.77 (0.32)	17
Idarubicin	1	500 (6.03)	60 (0.72)	12
Gemcitabine	1	5500 (66.35)	275 (3.32)	5
Bortezomib	1	1500 (18.10)	0	0
Carboplatin	2	4600 (55.49)	0	0
Dactinomycin	2	1780 (21.47)	0	0
Ifosfamide	1	644 (7.77)	0	0

Strategies to Curb Wastage

Among the total 140 drug prescriptions, measures to curb wastage were observed in 27 (19.29%) of the administrations. Measures such as sharing of vials were adopted in five (3.57%) of the administrations, while rounding was done in 22 (15.71%) of them.

Comparison of Drug Doses with Available Vial Sizes for Each Drug

When the doses of drugs prescribed to the vial strength were available at our setup (as shown in Table 6), the highest frequency of dose matching the vial size was seen for L-asparaginase. For 31 L-asparaginase administrations, the dose perfectly matched the vial size available in five cases (10,000 IU). However, for drugs with high wastage, such as doxorubicin, the median dose prescribed in our study, i.e., 33.9 mg (range 16-48), did not match the vial strengths available at our hospital (10, 50 mg).

**Table 6:** Details of available vial strengths and matching with prescribed dose (n=140) *: Drug amount in IU; NA: Not applicable, as all patients received a varying dose; Total number of drug administration, n=140

Drug name	Frequency of drug administration (n)	Commonest dose prescribed (mg)	Median dose prescribed mg(range)	Frequency of vials matching prescription dose(n)	Formulation available in our setup (mg)	Formulation available in the Indian market (mg)
L-Asparaginase*	31	10000	6800 (4000-10000)	5	5000, 10000	5000, 10000
Vincristine	29	1.5	1.3 (0.5-2.25)	2	1	1, 2
Cyclophosphamide	6	NA	600 (150-2068)	1	500, 1000	50, 100, 200, 500, 1000
Methotrexate (IV)	5	2820	1410 (1000-2820)	1	500	25, 50, 1000
Cytarabine	8	NA	63 (37.5-80)	0	100, 1000	100, 500, 1000
Doxorubicin	6	16	33.9 (16-48)	0	10, 50	10, 20, 50, 200
Daunorubicin	8	14	14 (13.7-21)	0	20	20, 50, 200
Methotrexate (IT)	26	12	12 (8-12)	0	15	15

## Discussion

Distress financing pays for most hospitalizations for paediatric cancer care patients in both rural (over 60%) and urban (40%) areas [10]. Given the enormous economic burden posed by anticancer drug treatment, it is unacceptable to bear the incremental cost of drug wastage. In this study, we audited parenteral chemotherapy drug wastage and estimated the economic loss incurred due to it at our paediatric oncology daycare unit.

Our study evaluated 100 patients who received 140 parenteral drug administrations of 21 different chemotherapy drugs. We found that 19.61% of the parenteral chemotherapy drug was wasted, which is similar to the study conducted by Gopisankar et al. [6]. In their study, they prospectively quantified chemotherapy drug wastage in adult patients and observed significant drug wastage of 19.72% in three months and 17.14% in one year for 313 patients attending the oncology unit. Several other studies conducted in oncology units have reported significant, but highly variable, drug wastage ranging from 1% to 33.8% [11-15]. However, most of the reported studies were conducted on adult cancer patients, and there is sparse data on paediatric patients.

In our study, we observed that, the amount of wasted drugs, i.e., 19.61%, resulted in an economic loss of 31,929.95 INR (385.19 USD), which accounted for 28.98% of the total drug cost. Similarly, in a study conducted by Gopisankar et al. [6], the cost due to drug wastage was found to be 17.14% of the total expenditure on drugs over one year. In a drug waste study by D'Souza et al. [4], 6.1% of the reconstituted drugs were wasted, and the cost analysis amounted to 11.1% of the total drug cost. The lesser amount of wastage observed in D'Souza et al.'s study suggests that some strategies for waste reduction may be already in place in their setup, although this was not clarified in the study results.

We found that doxorubicin 112.2 mg (37.4%, 95% CI: 16.67-58.12) had the highest amount of drug wastage, followed by cytarabine 280 mg (35%, 95% CI: 0-62.5) and L-asparaginase 83,400 IU (27.34%, 95% CI: 20.27-33.52), which were prescribed for ALL. The economic loss due to this wastage was 3,301.36 INR (39.83 USD) for doxorubicin, 681 INR (8.22 USD) for cytarabine, and 12,054 INR (145.41 USD) for L asparaginase. Hence, the baseline cost of a single unit results in more financial loss for even smaller wastage, as seen with L asparaginase. Other drugs frequently used in the treatment of ALL, such as intrathecal methotrexate administration (n=26) and vincristine (n=29) administrations, also contributed to wastage of 23.08% and 18.63% and financial loss of 289.27 INR (3.49 USD) and 328 INR (3.96 USD), respectively. The individual drug wastage seen in our study cannot be compared to other studies as the majority of the reported studies were conducted in adult cancer patients with different drug use spectrums.

In our study, the drug wastage and financial loss encountered for the single administration of irinotecan prescribed for primitive neuroectodermal tumours and fludarabine prescribed for acute myeloid leukaemia were 85% and 68%, respectively. The financial loss for irinotecan was 3,305 INR (39.87 USD), and for fludarabine, it was 6,204 INR (74.84 USD). However, there was no drug wastage observed in bortezomib, carboplatin, dactinomycin, or ifosfamide administrations since the dose administered matched the amount available in the vial exactly.

Various mitigation approaches can significantly reduce chemotherapy drug wastage. For instance, a study by Fasola et al. [15] found that using multidose vials with stability for up to 24 hours, scheduling chemotherapy sessions by grouping patients as per pathology or drug, rounding by 5%, and using appropriate vial size as per the estimated daily usage of each drug reduced drug waste expenditure by 45% [16]. These cost and waste containment strategies have been proven effective and can be implemented to optimize resource utilization in cancer care. For commonly used drugs such as l-asparaginase, intrathecal methotrexate, and vincristine in ALL treatment, waste mitigation strategies such as pathology-wise batching or drug-wise batching can be considered. However, the cost-effectiveness of these approaches needs to be evaluated in further studies before implementation.

In vial sharing, the remainder from each vial is retained and can be used for the next patient, while dose rounding is a method that either increases or decreases a prescribed dose to the nearest whole vial strength available [17]. However, our study found that vial sharing and rounding of dose were used in only 19.29% (n=27) of drug administrations. Rounding was done in 15.71% (n=22), and sharing was done in 3.57% (n=5) of drug administrations. To ensure safety and effectiveness, each institution should establish its criteria for automatic dose rounding, allowable percentage, and processes for operationalizing and documenting any modifications to the original prescribed dose. Additionally, exceptions to the dose-rounding policy should be determined a priori [8].

It is worth noting that all drug formulations available at your pharmacy were single-dose vials, as per the manufacturer's label, because they lack preservatives. Therefore, each shared vial must be appropriately logged, unpackaged, and stored, and its sterility must be ensured for later use. As two or more patients are treated from a single vial, the chances of microbial contamination cannot be negated, and proper precautions must be taken to minimize the risk of infections [17]. Vial-sharing challenges can be eased through drug vial optimization (DVO), which extends drug sterility and stability up to seven days using closed-system drug transfer devices (CSTDs). These devices move drugs between containers, such as vials to syringes, without contamination or environmental release. In an international survey by Gilbar et al., only India and Japan among 12 countries denied using DVO for anticancer injections [8].

For doxorubicin, cytarabine, methotrexate (intrathecal), and vincristine, the prescribed doses did not align with the available vial sizes. Regarding l-asparaginase (n=31), only five drug administrations perfectly matched the vial sizes. Bach et al. [12] suggested that policymakers should urge manufacturers to offer drug packaging in various sizes to minimize wastage. Additionally, further research into disease-specific body surface areas and weights could provide insights into ideal vial size options for waste reduction. Establishing guidelines, such as limiting wastage to a specified percentage of the vial size based on average patient body surface area or weight derived from disease-specific population data, could incentivize pharmaceutical companies to package drugs in sizes that reduce excessive wastage [13].

The limitations of our study are that monoclonal antibodies and small molecules were not routinely used at our institute, which is unlike the private sector where they are now widely used. Additionally, to devise a comprehensive strategy to minimize drug wastage, it would be appropriate to estimate wastage encountered with specific cancers such as ALL in our setup.

## Conclusions

The amount of wasted drugs was 19.61%, which resulted in an economic loss of 31,929.95 INR (385.19 USD), which accounted for 28.98% of the total drug cost in this study. In the realm of paediatric oncology care, a holistic approach is essential to effectively address drug wastage and the associated financial implications. One strategy involves encouraging pharmaceutical manufacturers to tailor vial sizes to meet the unique needs of the paediatric population. Additionally, exploring the feasibility of multi-dose vials with extended stability could prove advantageous. Healthcare providers can also play a crucial role by developing institution-specific protocols for dose rounding and vial sharing, informed by the stability data provided in the package insert. This balanced approach can help optimize resources and improve the efficiency of paediatric oncology drug administration.
